# Spatial ecology of the *Neisseriaceae* family in the human oral cavity

**DOI:** 10.1128/spectrum.03275-24

**Published:** 2025-04-08

**Authors:** Jonathan J. Giacomini, Julian Torres-Morales, Floyd E. Dewhirst, Gary G. Borisy, Jessica L. Mark Welch

**Affiliations:** 1 ADA Forsyth Institute, Cambridge, Massachusetts, USA; 2 Harvard School of Dental Medicine 124048 , Boston, Massachusetts, USA; 3 Marine Biological Laboratory 42700, Woods Hole, Massachusetts, USA; University of the Pacific, Arthur A. Dugoni School of Dentistry, San Francisco, California, USA

**Keywords:** oral microbiome, metapangenomics, habitat specialization, microbial ecology, *Neisseriaceae*

## Abstract

The human oral microbiome is a diverse ecosystem in which bacterial species have evolved to occupy specific niches within the oral cavity. The *Neisseriaceae* family, which includes human oral species in the genera *Neisseria*, *Eikenella*, *Kingella*, and *Simonsiella*, plays a significant role in both commensal and pathogenic relationships. In this study, we investigate the distribution and functional adaptations of *Neisseriaceae* species across oral habitats, focusing on their site tropisms and ecological roles. We employed a metapangenomic approach in which a curated set of reference genomes representing *Neisseriaceae* diversity was used for competitive mapping of metagenomic reads. Our analysis revealed distinct habitat preferences among *Neisseriaceae* species, with *Kingella oralis*, *Neisseria elongata*, and *Neisseria mucosa* primarily found in dental plaque; *Neisseria subflava* on the tongue dorsum; and *Neisseria cinerea* in the keratinized gingiva. Functional enrichment analyses identified genes and pathways underpinning habitat-specific adaptations. Plaque specialists showed metabolic versatility, with adaptations in nitrogen metabolism, including nitrate reduction and denitrification, lysine degradation, and galactose metabolism. Tongue dorsum specialists exhibited adaptations including enhanced capabilities for amino acid biosynthesis, short-chain fatty acid and glycerol transport, as well as lipopolysaccharide glycosylation, which may aid in resisting antimicrobial peptides and maintaining membrane integrity. These findings provide insights into the ecological roles and adaptive strategies of *Neisseriaceae* species within the human oral microbiome and establish a foundation for exploring functional specialization and microbial interactions in these niches.

**IMPORTANCE:**

Unraveling the distribution and functional adaptations of *Neisseriaceae* within the human oral microbiome is essential for understanding the roles of these abundant and prevalent commensals in both health and disease. Through a metapangenomic approach, we uncovered distinct habitat preferences of various *Neisseriaceae* taxa across the oral cavity and identified key genetic traits that may drive their habitat specialization and role in host-microbe interactions. These insights enhance our understanding of the microbial dynamics that shape oral microbial ecology, offering potential pathways for advancing oral health research.

## INTRODUCTION

The mouth as a biome comprises multiple unique habitats, each harboring its own community of microorganisms. These include the tongue; buccal mucosa; tooth surfaces, both sub-gingival and supragingival; gums; palate; throat; and tonsils. Studies have shown that bacterial species exhibit differential abundance across these distinct habitats (1–6). These habitat preferences, combined with the effects of oral hygiene and dietary habits, shape the microbial communities found in each niche (7–9).

The *Neisseriaceae* family is an important, complex group of bacteria commonly found in the human oral cavity, respiratory tract, and other mucosal surfaces (10). Key genera such as *Neisseria*, *Eikenella*, *Kingella*, and *Simonsiella* are known to interact with host tissues and other microorganisms, playing roles in both commensal and pathogenic relationships. These genera are notable for their capacity to reduce nitrate, a crucial adaptation for survival in anaerobic environments, as well as for their ability to colonize host surfaces through adhesin-mediated mechanisms (11, 12). Understanding the genetic basis of these capabilities in the human oral cavity is essential for deciphering their ecological roles and may offer new insights into the functional traits that shape their distribution and interactions within the oral microbiome. Here we analyze these four genera together, as they are both functionally similar and phylogenetically closely related and, in some cases, nested within one another.

The genus *Neisseria* is known for *Neisseria meningitidis* and *Neisseria gonorrhoeae*, which cause meningitis and gonorrhea. However, much more abundant in the normal flora of the human oral cavity are several commensal species including *Neisseria bacilliformis*, *Neisseria cinerea*, *Neisseria elongata*, *Neisseria flavescens*, *Neisseria lactamica*, *Neisseria polysaccharea*, *Neisseria sicca*, and *Neisseria subflava*, many of which have been shown to colonize different sites within the human oral cavity (6). Studying the habitat distributions and genetic traits of these species may reveal functional adaptations that enable these species to thrive in specific oral microenvironments, highlighting potential genetic drivers of site-specific colonization.

The genus *Eikenella* contains five named species, including *Eikenella corrodens* and four newly isolated species, *Eikenella exigua*, *Eikenella glucosivorans*, *Eikenella halliae* and *Eikenella longiqua. Eikenella corrodens*, recognized for its characteristic “pit-forming” growth on agar plates (13), is a common inhabitant of the human mouth in both healthy individuals (5) and those with periodontitis (14). Other *Eikenella* spp. have been isolated from a variety of human sites (15, 16), but whether they are normal residents of the oral cavity has not yet been established.

The *Kingella* genus comprises four species known to inhabit the human microbiome, including *Kingella kingae*, *Kingella denitrificans*, *Kingella oralis*, and *Kingella negevensis. Kingella kingae* is a significant pathogen in pediatric patients (17) but is rarely detected in the oral cavity. Conversely, the commensal species *K. denitrificans and K. oralis* are common residents of the oral microbiome. Metagenomic analysis of 16S rRNA sequences indicates they predominantly inhabit dental plaque samples (5). It is not yet established whether *K. negevensis*, which has been cultivated from the oropharynx of healthy children (18), is a normal resident of the human oral cavity.

*Simonsiella* spp. are found in the oral cavities of various animals, although only a single species, *Simonsiella muelleri*, is found in humans. Unlike many other oral bacteria, *Simonsiella* spp. divide longitudinally rather than end-to-end, thereby forming large, filamentous structures that aid in their attachment to epithelial cells (19).

Despite the ecological importance of *Neisseriaceae*, the extent to which these bacteria exhibit site-specific adaptations within the oral cavity is not well understood. In this study, we aim to investigate the distribution of *Neisseriaceae* species across different oral habitats and identify candidate genetic and metabolic pathways that contribute to their site-specific adaptations. To achieve this goal, we employ a metapangenomic approach, which combines the strengths of pangenomics and metagenomics (1–4, 20). By mapping whole-genome shotgun metagenomic data to a pangenome, we analyze the relative abundance of *Neisseriaceae* taxa and explore the distribution of specific genes and pathways that may drive their niche specialization.

## MATERIALS AND METHODS

The following analyses were conducted primarily using Anvi’o (v.8) (21) and Python (v.3.7.9) (15). Figures were generated using R (v.4.1.2) (16).

### Reference genome collection

To create a reference genome collection representing the diversity of *Neisseriaceae* in the human oral microbiome, we obtained publicly available RefSeq genomes from the National Center for Biotechnology Information (NCBI) database (downloaded on 21 September 2023) for *Neisseria*, *Eikenella*, *Kingella*, and *Simonsiella* spp. We used the NCBI data set program to retrieve metadata and FTP links used to download the genomes. In total, we downloaded 3,937 genomes, of which 2,262 were *N. meningitidis*; 1,055 were *N. gonorrhoeae*; and 620 were other *Neisseria*, *Eikenella*, *Kingella*, and *Simonsiella* taxa. Details about the genomes, including their genus, species, strain, BioSample, BioProject, isolation host, isolation site, RefSeq status, type strain, disease association, and submitter ID can be found in the supplemental data (Table S1).

We then performed quality control and dereplication steps to ensure that each genome in the collection had a completeness of at least 90%, had a contamination level below 5% as estimated by CheckM2 (22) (see supplemental data; Table S2), and had no more than 98% average nucleotide identity (ANI) with any other genome (see supplemental data; Table S3). This process resulted in a set of 213 high-quality reference genomes representing the diversity of *Neisseriaceae* found in the human microbiome.

### Pangenome construction

Using Anvi’o, we constructed a pangenome following previously developed methods (1, 3, 4, 20). First, we used *anvi-script-reformat-fasta* to replace non-canonical nucleotide letters with “N” and remove from each reference genome all contigs with length less than 300 nt. We then converted each genome into an Anvi’o-compatible contig database using *anvi-gen-contigs-db*. Open reading frames, hereafter referred to as genes, were identified by Prodigal (v.2.6.3). Functional annotation of genes was achieved using multiple Anvi’o scripts, including *anvi-run-hmms* to find bacterial single-copy genes (Bacteria71 SCG set) (23, 24) with hidden Markov mode profiles, *anvi-run-ncbi-cogs* using blastp (v.2.10.1+) to annotate with the cluster of orthologous genes (COGs) database (v.COG20) (25), and *anvi-run-pfams* and *anvi-run-kegg-kofams* with hmmscan from HMMER (v.3.3.1) (26) to functionally annotate with Pfams (v.34.0) (27) and KOfams/KEGG Modules (v.97.0) (28), respectively. We then used *anvi-pan-genome* to construct the annotated pangenome using blastp to calculate the amino acid-level identity between all possible gene pairs, with weak matches removed using the minbit criterion of 0.5. The *anvi-pan-genome* program uses a Markov cluster algorithm to group genes into putatively homologous groups called “gene clusters.” We set the mcl-inflation parameter to 10, as suggested by Anvi’o, for comparing very closely related genomes (https://merenlab.org/2016/11/08/pangenomics-v2/). Amino acid sequences within gene clusters were aligned with MUSCLE (v.3.8.1551) (29). Finally, we performed hierarchical clustering across gene clusters and genomes using Euclidean distance and Ward linkage. This resulted in a pangenome showing the distribution of core and accessory genes across the reference genomes.

### Phylogeny, average nucleotide identity, and comparison with Genome Taxonomy Database

We constructed a phylogenetic tree based on the amino acid sequences of 71 bacterial single-copy core genes (23, 24). We first used the Anvi’o program *anvi-get-sequences-for-hmm-hits* to align protein sequences using MUSCLE (v.3.8.1551) (29), concatenate gene sequences, return only the most significant hit, and output amino acid sequences. Only genes that occurred in at least 50% of the genomes were used for the analysis, which in this case included all 71 genes. We trimmed alignments using trimAl (30) with the setting ‘-gt 0.5’ to remove all positions that were gaps in more than 50% of sequences. Maximum likelihood phylogenetic trees were then computed using IQ-TREE (31) with the WAG model (32) and bootstrap replicate support of 1,000. We included a type strain genome for *Burkholderia cepacia* (strain BC16, GCA_009586235.1) to root the trees. To estimate pairwise whole-genome ANI between the selected reference genomes in the pangenome, we used the Anvi’o program *anvi-compute-genome-similarity* with the parameters ‘--program pyANI’ and ‘--method ANIb’. To compare genomes against the classification in Genome Taxonomy Database (GTDB), we used GTDB-Tk (v.2.3.0) (33) with classify_wf and the R214 reference data release.

We used a multifaceted approach to identify sub-species-level genetic clades, hereafter referred to as sub-groups, within the *N. subflava* and the *N. mucosa* major clades. Our methods included assessing ANI and two single-copy core gene phylogenies: one based on the universal 71 bacterial single-copy core genes (SCCGs) and the other on SCCGs extracted from the pangenome for the respective focal genomes. For each species, we constructed three tanglegrams: one comparing the ANI-based hierarchical clustering tree with the 71 bacterial SCCGs, one comparing the ANI-based hierarchical clustering tree with the pangenome SCCGs, and one comparing the 71 bacterial SCCGs with the pangenome SCCGs. For ANI, we used the gap statistic in conjunction with clusGap in the R package “cluster” (v.2.1.4) (34), which calculates a goodness-of-clustering measure (the “gap” statistic) for each possible number of *k* clusters. We also used the fviz_nbclust function in the R package “factoextra” (v.1.0.7) (35) to determine and visualize the optimal number of clusters using within-cluster sums of squares for each possible number of *k* clusters.

### Distribution of *Neisseriaceae* genomes across human oral sites

We analyzed the distribution of natural populations of *Neisseria*, *Eikenella*, *Kingella*, and *Simonsiella* taxa across human oral sites by mapping shotgun metagenomic sequences from the National Institutes of Health Human Microbiome Project (HMP) (36, 37) to our curated set of reference genomes. To obtain data from the HMP portal (https://portal.hmpdacc.org/), we searched for metagenomes using the following parameters: oral sites (buccal *mucosa*, supragingival plaque (SUPP), sub-gingival plaque, dorsum of tongue, hard palate, palatine tonsil, throat, and saliva), Healthy Human Study, fastq files (FASTQ), and whole-genome sequencing (wgs_raw_seq_set). The metagenomes consisted of ~100 bp paired-end reads that were sequenced from samples collected from nine oral sites in phases 1 and 2 of the HMP. We performed quality filtering using *iu-filter-quality-minoche* (38), which is based on recommendations from Minoche et al. (39) for Illumina sequencing data. This resulted in a total of 2.5 billion quality-filtered metagenomic short reads from 1,297 samples across nine different oral sites, including three main sites in the oral cavity with large sample sizes (the buccal *mucosa* [*n* = 378], supragingival plaque [*n* = 398], and tongue dorsum (TD) [*n* = 428]) and six other sites with smaller sample sizes (sub-gingival plaque [*n* = 24], keratinized gingiva (KG) [*n* = 17], hard palate [*n* = 1], palatine tonsil [*n* = 25], throat [*n* = 18], and saliva [*n* = 8]). Details about the metagenomes, such as their BioSample ID, download links, and quality filtering results can be found in the supplemental data (Table S4).

We competitively mapped individual qualityfiltered metagenomic samples to a concatenated file of the selected reference genomes in the pangenome using bowtie2 (v.2.4.1) (40) with the “--very-sensitive,” “--end-to-end,” and “--no-unal” flags. Competitive mapping assigns each mapped read to a single genome that provides the best match. BAM files were generated from the read alignment data using Samtools (v.1.9) (41), and the Anvi’o program *anvi-single-profile* was used to create a profile database containing coverage data for each metagenome. Profiles were then merged for each oral site using *anvi-merge*. We then extracted the mean depth of coverage and breadth of coverage for reads aligned to each genome using *anvi-summarize*. We classified a genome as detected in a metagenomic sample when breadth of coverage was at least 50%. We set a 50% breadth of coverage threshold to ensure robust detection of genomes in each metagenomic sample, focusing on well-represented taxa while excluding low-abundance or poorly covered genomes. We then calculated the relative abundance of each detected genome by averaging its depth of coverage across nucleotide positions in which coverage was within the interquartile range (Q2Q3) and dividing by the total mean depth of Q2Q3 coverage for all reference genomes. We used the Q2Q3 quartiles of the mean depth of coverage to filter out outliers in coverage caused by mobile elements or other highly similar sequences shared among different taxa in the community.

### Classification of habitat preferences

We adapted a previously developed classification algorithm (5) to associate each species or sub-group with one or more sampling sites where that clade is most differentially abundant or prevalent. Briefly, the algorithm creates a list of all possible binary mappings of eight sampling sites (buccal mucosa [BM], KG, PT, SUBP, SUPP, SV, TD, and TH) to generate all two-group combinations of sampling sites. It then tests whether the central tendencies of group 1 and group 2 differ significantly. For the abundance metric, we summed the mean Q2Q3 coverage for each genome within a species’ clade and used Student’s *t*-test to calculate the *T* statistic for each map. For the prevalence metric, we selected the maximum breadth of coverage value from the set of genomes for each species’ clade and estimated a binary detection value for each sample based on a 50% breadth of coverage threshold. We then modeled prevalence using a generalized linear model fit with a binomial distribution to calculate the Rao statistic for each map. For both the abundance- and prevalence-based classification methods, the map that yielded the largest absolute test statistic was used to associate the species clade with the sampling site(s).

### Functional analysis of detected *Neisseriaceae* genomes across human oral sites

Metabolic capabilities of genomes were predicted using the Anvi’o script anvi-estimate-metabolism (with parameters: --kegg-output-modes modules). The metabolic pathways considered by this script are those outlined in the KEGG Module Database and defined by KEGG Orthologs (KOs) (28). Each KO represents a specific gene function, and a KEGG module is a collection of KOs that work together to complete the steps of a metabolic pathway. We used the default completion threshold of 0.75, which scores a metabolic pathway as “complete” within a genome when at least 75% of the enzymes in the pathway are present in the genome. We then used the Anvi’o script anvi-compute-metabolic-enrichment to identify complete metabolic pathways differentially enriched in one set of genomes compared to another based on their habitat preferences.

To identify COG functional annotations that are differentially enriched or depleted in one set of genomes compared to another, we used the Anvi’o script anvi-compute-functional-enrichment. The script associated each gene cluster with the most frequently annotated function and generated a frequency table of functions across genomes. An enrichment test was then conducted using a generalized linear model with a logit linkage function to obtain the enrichment score and an adjusted *P* value (*q* value). For both the KEGG and COG analyses, we considered modules or functions to be significantly enriched if they had a *q* value of less than 0.01.

## RESULTS

### Pangenome, phylogeny, average nucleotide identity, and comparison with GTDB

**Fig 1:**
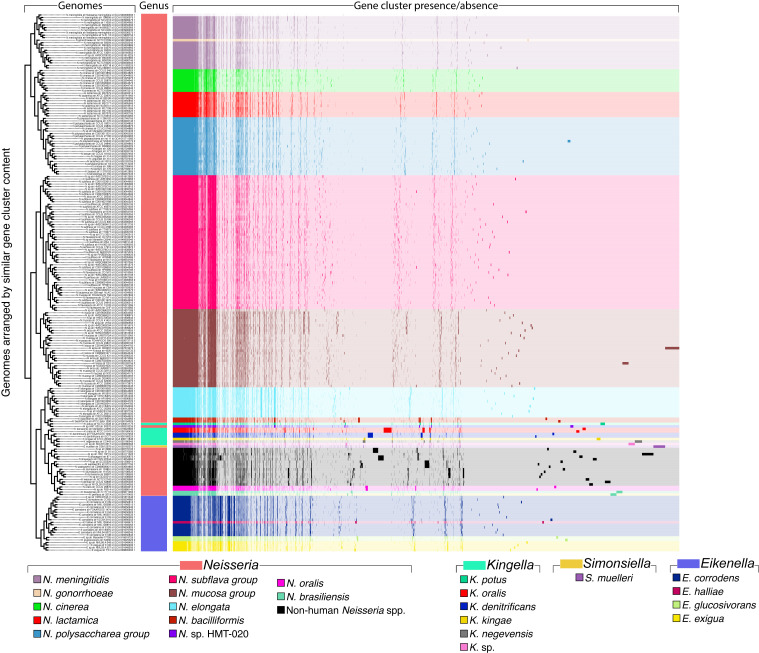
Human-associated *Neisseriaceae* species pangenome constructed from (*n* = 213) NCBI RefSeq genomes. For all genomes, open reading frames were predicted, and NCBI blastp was used to calculate amino acid sequence similarity between all possible gene pairs. A Markov cluster algorithm was used to cluster similar sequences to identify homologous genes (i.e., gene clusters). Gene clusters are colored by species and arranged based on their presence or absence across the genomes. Genomes are hierarchically clustered based on gene cluster frequency (i.e., the number of representatives of each gene cluster present in each genome), shown by the dendrogram on the left. This pangenomic analysis results in distinct groups by species.

**Fig 2:**
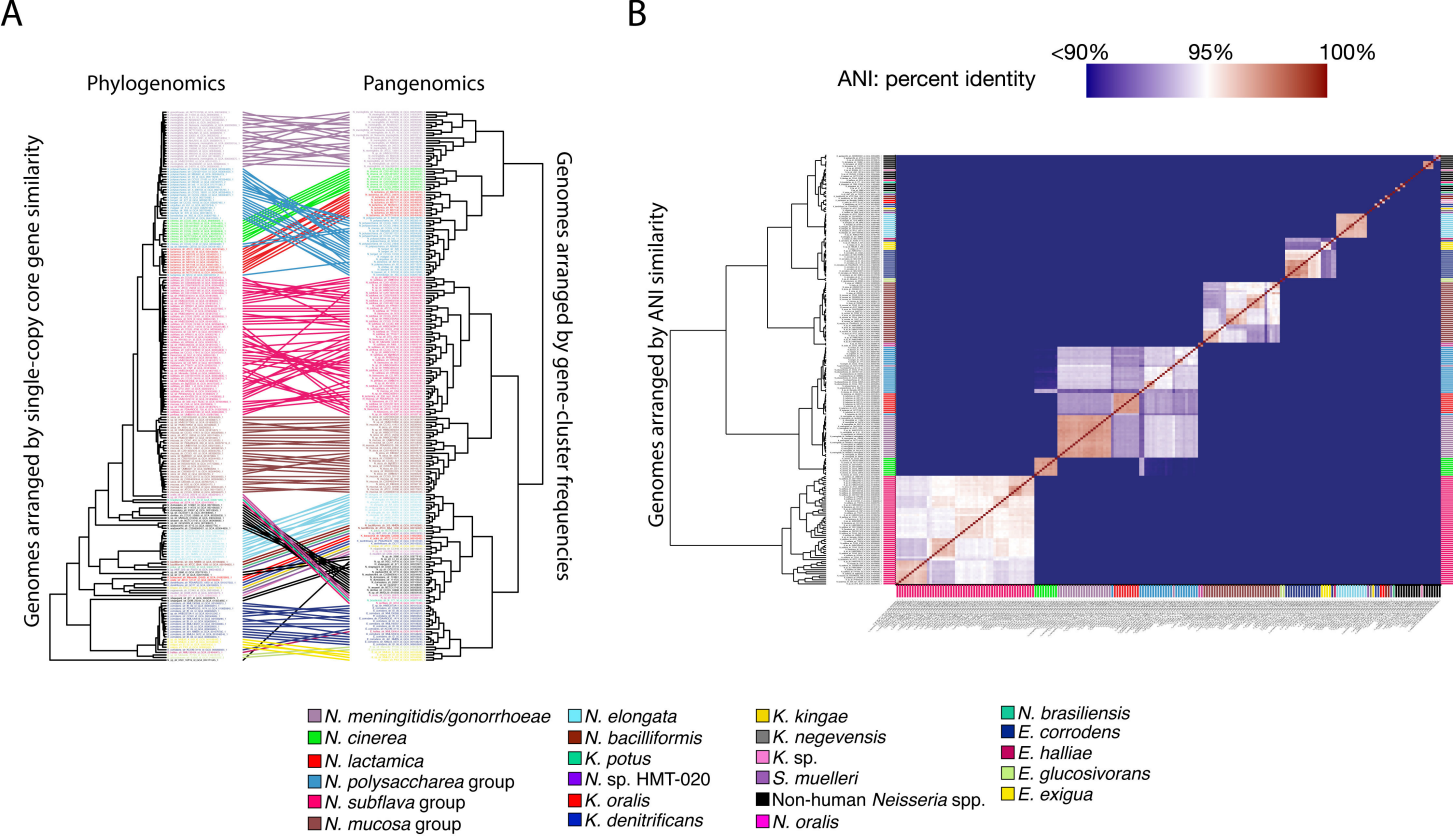
(A) Phylogenomic and pangenomic tree comparison of *Neisseriaceae* reference genomes clusters them into the same species-level groups. Rectangle color indicates species. The phylogenomic tree was constructed using maximum likelihood with concatenated single-copy core genes. The pangenomic tree was constructed using the gene cluster frequencies present in each genome. Lines connect rectangles that represent the same genome. (B) Average nucleotide identity (ANI) comparison of *Neisseriaceae* reference genomes included in the pangenome. ANI represents the genome-level similarity at the nucleotide level between any two genomes and reveals distinct species, corroborating the results of the pangenome clustering. The rectangle color indicates species as in panel A. The color scale denotes genome percent similarity; 100% is red; 95% is white; 90% and below are blue.

Accurate placement of bacterial genomes into biologically meaningful groups is crucial for identifying genes and functions that drive habitat specialization in the oral cavity. Our pangenomic analysis of whole-genome sequences identified genetic clades largely consistent with phylogeneticbased taxonomy, including NCBI and GTDB classifications (Fig. 1; supplemental data, Table S5). When compared with phylogenomics and ANI, the pangenomic clades showed strong agreement (Fig. 2A and B), though some genome arrangements differed between methods, highlighting the value of using multiple independent measures of relatedness.

Overall, our analysis showed that genera and species generally grouped according to existing taxon naming, though there were some notable exceptions. In *Neisseria*, the clades for both *N. subflava* and *N. mucosa* included genomes from multiple named species, forming distinct sub-groups with clear genetic differences as indicated by ANI values (Fig. S1 and S2; supplemental data, Table S6). Additionally, *N. polysaccharea* formed a distinct clade with *Neisseria bergeri*, *Neisseria urigultitaei*, *Neisseria maigaei*, *Neisseria viridiae*, *Neisseria blantyrii*, *Neisseria benedictiae*, and *Neisseria basseii* in our analysis, consistent with previous findings that *N. polysaccharea* includes multiple phylogenetic clusters requiring reclassification (42). Notably, this clade was most closely related to *N. lactamica*, *N. meningitidis*, and *N. gonorrhoeae*, which are well-studied human-associated species. One interesting finding is that *N. cinerea*, an oral resident, clustered most closely with this group, suggesting possible evolutionary or ecological links. *Eikenella* species formed a distinct group, supporting the classification of *E. corrodens*, *E. halliae*, *E. exigua*, and *E. glucosivorans* as separate species. *Kingella* also largely formed a coherent group, but *Kingella potus* was an exception, grouping more closely with *N. bacilliformis* and *Neisseria* sp. HMT-020 than with other *Kingella* species (Fig. 2B; supplemental data, Table S7). Additionally, *Simonsiella muelleri* was nested within the *Kingella* genus, suggesting the need for a re-evaluation of the taxonomy for this genus. These results underscore the complexity of *Neisseria* phylogeny and taxonomy, with implications for understanding both ecological specialization and evolutionary relationships. Further details of these findings are provided in the supplemental text.

### Distribution of *Neisseriaceae* genomes across human oral sites

**Fig 3:**
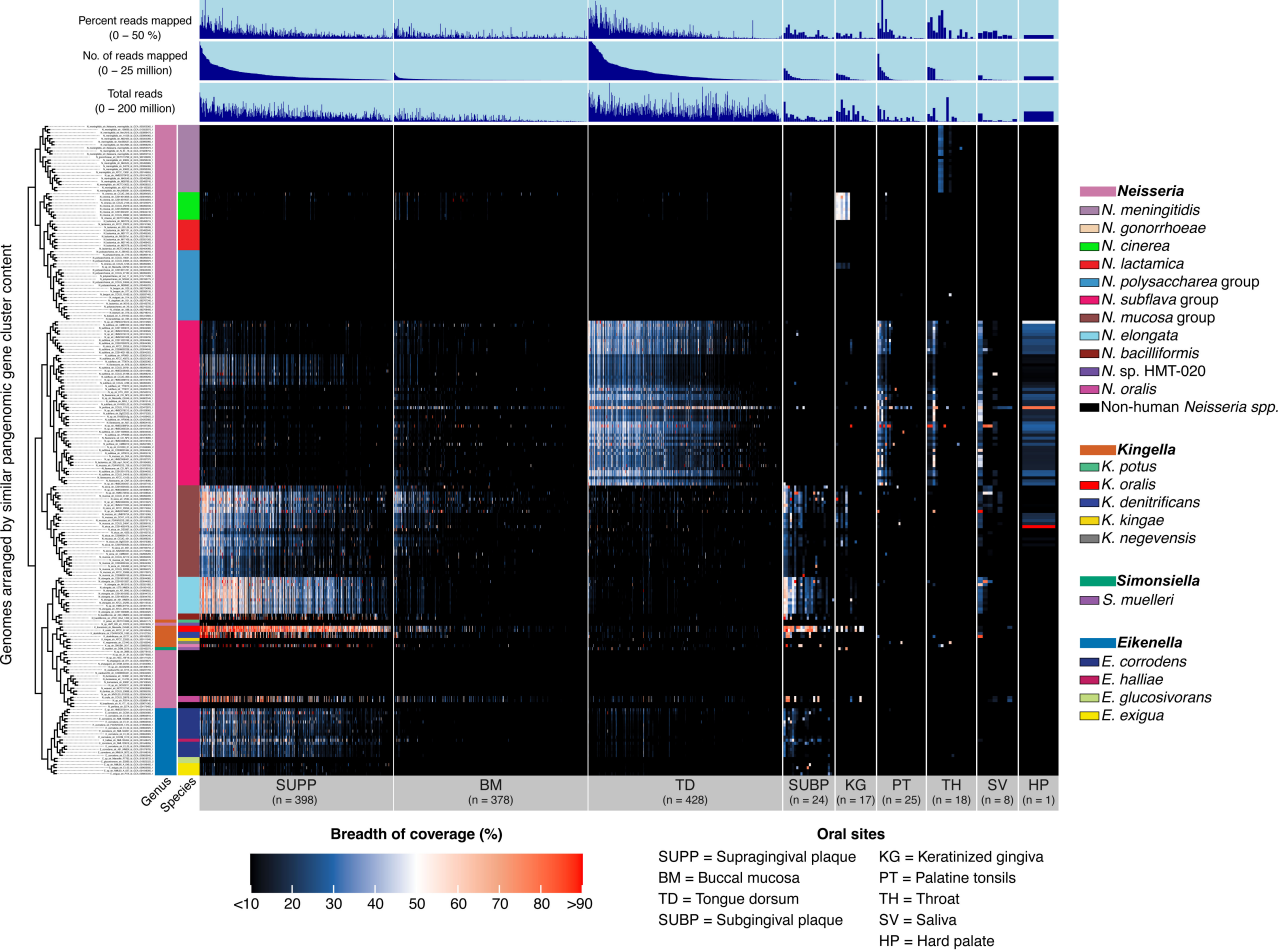
Genome breadth of coverage heatmap of *Neisseriaceae* reference genomes for 1,297 Human Microbiome Project metagenomic samples from nine major oral sites. This heatmap highlights genome coverage across samples, with broader coverage indicating a higher likelihood of detecting the genome at a specific site. Each row displays the proportion or breadth of coverage of a genome across all samples. Samples are ordered within each oral site by the decreasing number of reads mapped to the set of genomes. From left to right, oral sites are supragingival plaque (SUPP, *n* = 398), buccal mucosa (BM, *n* = 378), tongue dorsum (TD, *n* = 428 samples), sub-gingival plaque (SUBP, *n* = 24), keratinized gingiva (KG, *n* = 17), palatine tonsil (PT, *n* = 25), throat (TH, *n* = 18), saliva (SV, *n* = 8), and hard palate (HP, *n* = 1). For clearer visualization, we inflated the width of oral sites with small sample sizes. Additional data are shown for “total reads” in each sample, the number of “reads mapped” per sample, and “percent reads mapped” per sample.

**Fig 4:**
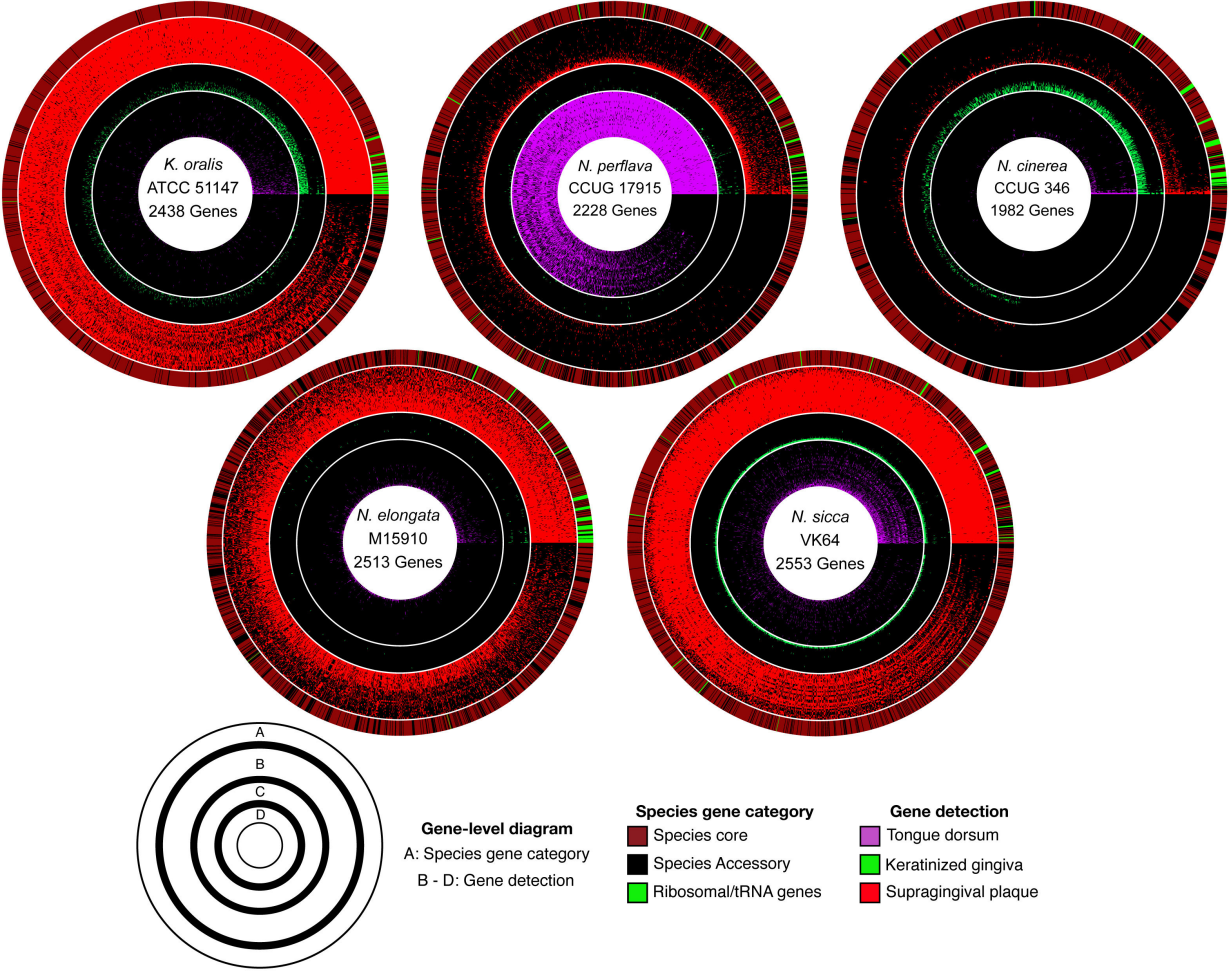
Gene-level detection diagrams for select *Neisseriaceae* genomes illustrate patterns of site specialization. Here, we display radial gene-level detection maps for five genomes showing the detection of genes across a subset of the top 30 metagenomic samples ranked by median coverage from three oral sites (supragingival plaque [red], keratinized gingiva [green], and tongue dorsum [magenta]). A gene was classified as detected within an oral site (colored red, green, or magenta, respectively) when at least 90% of the nucleotides of the gene had at least 1× coverage. A stringent 90% gene coverage threshold was used to ensure reliability of the detection metric. Genes are ordered according to their detection frequencies, and samples within each oral site are ordered ascending inward to outward based on the proportion of genes detected. The outermost layer for each diagram indicates whether a gene was core (brick red) or accessory (black) for each respective species. A group of ribosomal genes translated using hidden Markov models via the Anvi’o program anvi-run-hmms are indicated in bright green. Species and strain IDs were from the NCBI RefSeq database.

Understanding the distribution of *Neisseriaceae* across different oral sites is crucial for linking microbial functional potential to ecological niches. Based on metapangenomic mapping and determination of genomic breadth of coverage, we found that the genomes of *Neisseriaceae* taxa, organized by similar gene content, exhibited distinctive distributions among oral sites. (Fig. 3; supplemental data, Table S9). Several taxa exhibited high prevalence in certain habitats, including *N. mucosa*, *K. oralis*, and *N. elongata* in dental plaque, *N. subflava* on the tongue dorsum, and *N. cinerea* on the keratinized gingiva. Complementing these genome-level findings, we also generated gene-detection maps, which revealed that most genes were detected predominantly at the same sites where their corresponding genomes were prevalent (Fig. 4). The gene-level results confirmed the site tropisms suggested by whole-genome coverage and indicate that the gene content of sequenced genomes from cultivated strains is representative of the gene content in populations in the mouth. A few genes were detected across multiple habitats and were annotated with highly conserved functions or as transposable elements, likely reflecting cross-mapping between taxa with high sequence similarity or shared horizontally acquired elements.

The genomes of several other species were detected predominantly in dental plaque sites and sporadically in buccal mucosa and tongue dorsum samples, including *N. oralis*, *N. bacilliformis*, *E. corrodens*, *E. halliae*, *K. denitrificans* and *Kingella* sp. (strain SNUBH_2017). Genomes for *Eikenella exigua* and *Neisseria* sp. HMT-020 were rare in the human oral cavity, detected in only 5 out of 422 dental plaque samples. *Kingella negevensis* and *K. kingae* were similarly rare, the former detected in only a single palatine tonsil sample and a single throat sample, and the latter detected in only a single tongue dorsum sample.

The genomes of the pathogens *N. meningitidis* and *N. gonorrhoeae*, as well as their closely related commensals, *N. polysaccharea*, *N. lactamica*, and the recently named *N. bergeri*, *N. maigaei*, *N. uirgultaei*, *N. viridiae*, *N. blantyrii*, *N. basseii*, and *N. benedictiae*, were not detected in the healthy human oral cavity. Similarly, genomes from non-human-associated *Neisseria* species were not detected across all oral sites. Additionally, a single *Neisseria perflava* genome (strain 327A) that clustered with a *Neisseria brasiliensis* genome in the pangenome, and phylogeny was not detected in any samples, indicating a strong likelihood of species misclassification in NCBI.

**Fig 5:**
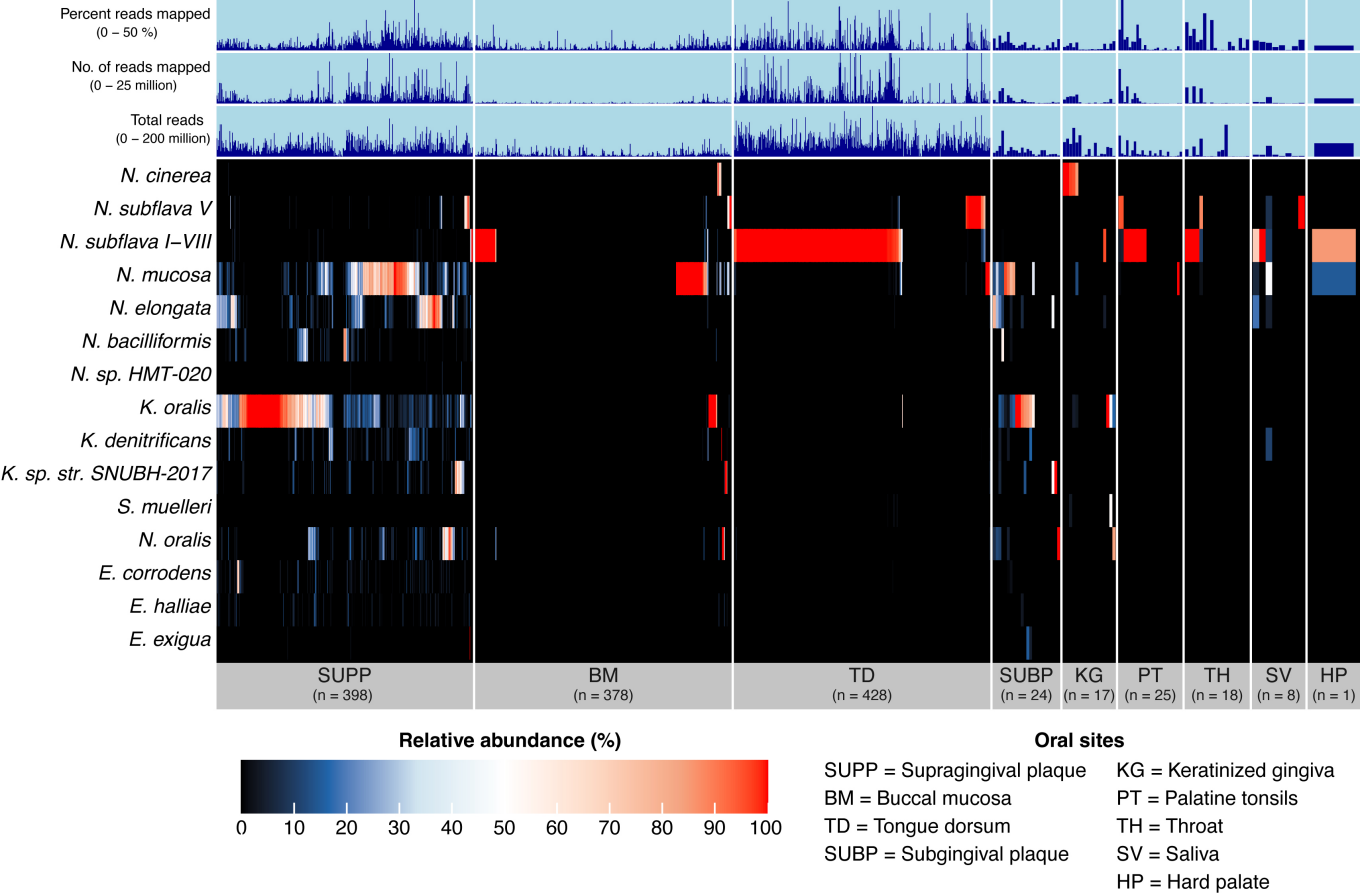
Heatmap displaying the relative abundance of *Neisseriaceae* species across nine major oral sites: supragingival plaque (SUPP), buccal mucosa (BM), tongue dorsum (TD), sub-gingival plaque (SUBP), keratinized gingiva (KG), palatine tonsils (PT), throat (TH), saliva (SV), and hard palate (HP). Rows represent individual species, while columns correspond to Human Microbiome Project metagenomic samples. The relative abundance of each species is calculated as the sum of relative abundances for all reference genomes within that species, based on the mean depth of coverage across nucleotide positions in the second and third quartiles (Q2Q3 interquartile range), after ranking nucleotides by depth of coverage. This value is then divided by the total mean coverage of all genomes within a sample. If a genome was undetected (i.e., less than 50% of nucleotides covered at 1×), its coverage was set to zero. Species (*y*-axis) and samples within oral sites (*x*-axis) are hierarchically clustered based on Bray-Curtis distances. To improve visualization, the width of oral sites with small sample sizes has been inflated.

Analysis of the relative abundance of species and sub-groups showed that while many dental plaque samples contained multiple detected species of *Neisseriaceae*, some plaque samples and most samples from mucosal sites contained only a single *Neisseriaceae* species, as indicated by the red bars in Fig. 5. The presence of only a single species per sample could arise from multiple causes. In some cases (e.g., *N. subflava* on the tongue and *N. cinerea* in keratinized gingiva samples), this single species was in high abundance, as indicated by the number of mapped reads in Fig. 5, suggesting that it is a significant member of the biota in that habitat. In other cases, such as in the BM, the number of mapped *Neisseriaceae* reads was generally low. In most such samples, either no *Neisseriaceae* taxon or only one of the species *N. subflava*, *N. mucosa*, or *K. oralis* crossed the detection threshold. These species are typically more abundant in the TD or SUPP and may be transient or low-abundance colonists of the rapidly exfoliating and generally thinly colonized buccal mucosa. In dental plaque (SUPP and SUBP), multiple species were frequently detected, including *N. mucosa*, *K. oralis*, or *N. elongata* and several lower-abundance taxa (Fig. 5). These species sometimes coexisted, as indicated by the blue and white bars in Fig. 5, while sometimes only one was present, as indicated by the red bars. Patterns of apparent mutual exclusion or coexistence highlight the complex ecological dynamics of *Neisseriaceae* species in the oral cavity and suggest that both competitive interactions and niche partitioning may contribute to their varied distribution across oral sites.

We determined habitat preference objectively by our multisite habitat classification algorithm, which is based on both the breadth and depth of genome coverage across samples, thus providing a more comprehensive view of habitat specialization than detection alone. The algorithm showed that each *Neisseriaceae* species predominantly inhabits one of three major oral habitats: dental plaque; the keratinized gingiva; or the set of sites including the tongue dorsum, throat, tonsils, and saliva (supplemental data, Table S8). These results serve as the basis for the subsequent functional enrichment analyses, where we explore the ecological and genetic adaptations associated with each habitat preference. One exception, which is visible in Fig. 3, was *N. subflava*, where the major clades favored the tongue dorsum, but one sub-clade preferred supragingival plaque. All detected *Kingella* and *Eikenella* species, as well as most *Neisseria* species, showed a preference for dental plaque, while *N. subflava* preferred the tongue dorsum and *N. cinerea* preferred the keratinized gingiva. *Simonsiella muelleri*, previously observed in high abundance on the hard palate in about one-third of the samples (5, 43), was scored as preferring the keratinized gingiva in this data set, as only a single hard palate sample was available.

### Functional analysis of *Neisseriaceae* genomes across human oral sites

Understanding the genetic basis of habitat specialization is key to unraveling how *Neisseriaceae* species thrive in distinct oral sites. To uncover the genetic drivers of specialization, we conducted enrichment analyses to identify functional annotations of genes that were statistically enriched in genomes with specific habitat preferences. We first performed a broad-level analysis comparing KEGG metabolic pathway completeness and COG functional annotations of detected genomes grouped according to their respective habitat preference (keratinized gingiva, dental plaque, or tongue dorsum). KEGG analyses provide insight into metabolic capacity via analysis of completeness of modules, whereas the COG database provides a more comprehensive analysis as it contains additional genes and functions absent in the KEGG database.

Genera and species were distinguishable based on the metabolic functions they encode, as indicated by their complete KEGG modules (Fig. S3). Five metabolic modules were enriched only in genomes of dental plaque specialists, and an additional module was enriched in both dental plaque and keratinized gingiva specialists, but none of these modules were present in all plaque specialist genomes (supplemental data, Table S11). This differentiation highlights the diversity of metabolic strategies among *Neisseriaceae* taxa in dental plaque.

Nitrogen metabolism differentiated *Neisseriaceae* species with respect to specific oral niches. Denitrification from nitrate (KEGG M00529) was enriched in dental plaque specialists. Many dental plaque specialists possess the genes required for nitrate, nitrite, and nitric oxide reduction, including *Eikenella corrodens*, *E. halliae*, *Kingella denitrificans*, *Kingella* sp. SNUBH-2017, *Neisseria bacciliformis, N. elongata*, *N. mucosa*, and *N. oralis* (supplemental data, Tables S14 and S15). Exceptions included the highly prevalent dental plaque taxon *K. oralis* and some strains of *N. mucosa*, which lacked genes encoding nitrate reductase. The hard palate taxon *S. muelleri* had genes for complete denitrification from nitrate to nitrogen gas. By contrast, the tongue dorsum specialist *N. subflava* and the keratinized gingiva taxon *N. cinerea* lacked nitrate reduction but retained other nitrogen metabolism capabilities. Supporting this pattern of nitrate reduction by dental plaque *Neisseriaceae*, completeness of the molybdenum cofactor (MoCo) biosynthesis pathway (KEGG M00880) was also enriched in dental plaque specialists. COG functional enrichment analysis further indicated the enrichment of nine genes associated with molybdopterin biosynthesis in plaque specialists. Molybdopterin (MoCo) is an essential cofactor required for nitrate reductase activity. These results provide insights into how nitrogen metabolism pathways may contribute to niche specialization and oral nitrogen cycling among *Neisseriaceae* species.

The ability to metabolize a variety of nutrient sources may be a key factor in the adaptation of dental plaque specialists to the oral environment. Two examples are the degradation of lysine and the metabolism of galactose. All human-associated *Neisseriaceae* genomes possess the genes required to produce succinate from L-lysine, whereas all dental plaque specialists possess an additional gene for the amino acid racemase RacX that converts D-lysine to L-lysine. The ability to utilize both L-lysine and D-lysine can be advantageous for bacteria, especially when encountering diverse nutrient sources. Dental plaque specialists were enriched in two additional pathways, the nucleotide sugar biosynthesis, which converts galactose to UDP-galactose (KEGG M00554) and galactose degradation, and the Leloir pathway, which converts galactose to alpha-D-glucose-1P (KEGG M00632). These pathways enable bacteria to utilize D-galactose as carbon and energy sources and are exclusively enriched in the plaque specialists *K. oralis*, *N. elongata*, and *Neiserria* sp. HMT-020. Finally, *N. elongata* and *Neisseria* sp. HMT-020 have a complete pathway for the glyoxylate cycle (KEGG M00012), which enables bacteria to convert acetate or other simple carbon compounds into biosynthetic precursors like glucose. These results indicate that dental plaque specialists vary in their metabolic versatility, which may play a crucial role in enabling plaque specialists to adapt to the complex, competitive dental plaque environment.

Beyond these metabolic pathway completeness findings with KEGG, COG functional annotation enrichment analysis revealed additional functions enriched in dental plaque specialists. These include functions related to adhesion, antimicrobial resistance, metal ion tolerance, amino acid metabolism, membrane transport, and redox stress response, as well as a range of uncharacterized or predicted proteins that may contribute to their unique adaptation to the dental plaque environment (Fig. S4; supplemental data, Table S12). Enriched adhesion mechanisms include proteins involved in fimbriae and pili assembly, such as the fimbrial sub-unit ScuA/B, the P pilus assembly chaperone FimC/PapD, and the outer membrane usher proteins FimD/PapC. These proteins may enhance the ability of plaque specialists to attach to host surfaces and form biofilms. Several antimicrobial resistance-associated genes were enriched in plaque specialists, including a beta-lactamase PenP, which is present in *Kingella* sp. strain SNUBH, *N. elongata*, and *N. mucosa*, as well as a vancomycin resistance protein YoaR, detected exclusively in *E. corrodens* and *Kingella* sp. strain SNUBH genomes. Enriched functions related to metal ion tolerance include two genes involved in copper resistance, CutA and CutF, which likely contribute to the ability of plaque specialists to withstand the high levels of antibacterial copper ions that can be present in the oral environment (44). Enriched functions related to redox stress response include Cu/Zn superoxide dismutase (SodC) and hemerythrin. SodC neutralizes reactive oxygen species, while hemerythrin helps bind oxygen and maintain redox balance. Similar to *Streptococcus mutans*, which uses oxidative stress regulatory mechanisms to compete in biofilms (45), *Neisseriaceae* plaque specialists are facultative anaerobes or microaerophiles that may rely on these enriched functions to thrive in the oxidative environment of dental plaque.

In the sole tongue specialist, *N. subflava*, four KEGG metabolic pathways were enriched relative to dental plaque and keratinized gingiva specialists. These include three pathways for amino acid biosynthesis—histidine, ornithine, and proline (KEGG M00026, M00028, M00015)—and one pathway for glycogen degradation (KEGG M00855), facilitating the conversion of glycogen to glucose-6-phosphate. Functional enrichment analysis using COG annotations revealed four genes that were present in all tongue specialist genomes but absent from dental plaque genomes (Fig. S4; supplemental data, Table S12). These include a predicted restriction endonuclease from the Mrr-cat superfamily, which may play a role in defending against foreign DNA. Other enriched genes were SatP, GlpF, and RfaJ. SatP is a succinate-acetate transporter, which likely supports metabolic versatility by enabling the transport of important short-chain fatty acids that can serve as carbon sources under nutrient-limited conditions. GlpF is a glycerol uptake facilitator, suggesting that glycerol uptake might be important for the metabolic needs of tongue dorsum specialists. RfaJ is a lipopolysaccharide (LPS) biosynthesis protein involved in LPS glycosylation, indicating that LPS modification might be crucial for interaction with the host immune system or maintaining outer membrane integrity. In gram-negative bacteria, alterations to the LPS are essential for resisting cationic antimicrobial peptides (46). A multidrug efflux system pump (EmrE) was also significantly enriched in the tongue dorsum-associated *N. subflava* genomes. Efflux pumps like EmrE actively transport a variety of antimicrobial compounds out of the cell, conferring resistance to a diverse range of antibiotics (47). Overall, these enriched functions underscore the metabolic versatility and protective adaptations of tongue dorsum specialists, highlighting their capacity for *de novo* amino acid synthesis, nutrient acquisition, and defense against environmental stressors.

Comparing close relatives with different habitat preferences may provide a more sensitive method of detecting genes conferring success in each habitat. Therefore, we conducted a more focused functional enrichment analysis comparing the two closely related species, *N. mucosa* and *N. subflava*, which have distinct habitat preferences for dental plaque and the tongue dorsum, respectively. Overall, this analysis supported results from the broad habitat-level analysis, revealing the same differential functions as above and additional gene functions that may drive habitat specialization. Genes that were specific to *N. mucosa* but were absent from *N. subflava* code for functions suggesting specialized adaptations for nutrient acquisition, redox homeostasis, and environmental stress resilience in plaque specialists. They include NAD/NADP transhydrogenase sub-units (PntA and PntB), which help maintain redox balance by generating NADPH; DAHP synthase (AroGA), which catalyzes the first step in aromatic amino acid biosynthesis; uroporphyrinogen-III synthase (HemD), involved in heme biosynthesis; beta-hydroxyacid dehydrogenase (MmsB), important for metabolizing certain intermediates; O-methyltransferase (YktD), involved in polyketide biosynthesis, important in microbial competition; and tellurite resistance protein (TehA). Conversely, genes present in *N. subflava* that are absent in *N. mucosa* code for functions that may reflect adaptations to the unique oxidative and nutrient conditions of the tongue dorsum environment. They include cytochrome b561 (CybB), involved in electron transport and potentially oxidative stress defense; ADP-ribosylglycohydrolase (DraG), which may play a role in cellular regulation; an outer membrane channel-forming protein (BP26), potentially facilitating nutrient uptake; and several uncharacterized membrane proteins. These differential functions were all from COG analysis; analysis of KEGG pathways showed no pathways differentiating *N. subflava* from *N. mucosa* (supplemental data, Table S16).

Despite the differing environmental conditions of KG, KEGG metabolic pathway enrichment analysis revealed the KG specialists *N. cinerea* spp. had a surprising overlap in their metabolic capabilities compared to *Neisseriaceae* taxa in tongue and dental plaque sites. Only a single COG function was enriched in KG specialists, a Na+/H+-dicarboxylate symporter (GltP). Such symporters are membrane proteins that mediate the intake of a wide variety of molecules with the concomitant uptake of sodium ions. These results indicate that KG specialists may rely on efficient nutrient uptake systems, like GltP, to adapt to the specific nutrient availability in the keratinized gingiva environment. *Simonsiella muelleri*, identified here as a keratinized gingiva specialist despite being a putative hard palate specialist, possesses glycerol kinase (GlpK), which is absent in all dental plaque and tongue specialists. GlpK plays a key role in the regulation of glycerol uptake and metabolism. However, the overall limited differences in gene content preclude strong conclusions on the functional differences that contribute to keratinized gingiva niche specialization.

## DISCUSSION

A growing body of research supports the idea that highly specialized microbial communities are adapted to distinct niches within the human mouth such as dental plaque, the tongue dorsum, and the keratinized gingiva (1–4, 20). Our findings align with previous research showing habitat tropisms of *Neisseria* spp. in the oral cavity (5, 6) and extend our understanding of such tropisms to include *Kingella*, *Eikenella*, and *Simonsiella* spp. Additionally, our functional pathway analysis highlights specific genes and metabolic pathways that may enhance survival and colonization in these environments.

Reduction of nitrate to nitrite plays a crucial role in the nitrogen cycle with significant implications for microbial ecology and human health. Our study revealed that nitrate reduction, as performed by *Neisseriaceae* taxa in the human oral cavity, is exclusive to dental plaque specialists. All genetic clades of the tongue dorsum specialist, *N. subflava*, including genomes from *N. flavescens*, lacked the NarGHIJ operon, suggesting they are incapable of nitrate reduction. Nitrate reduction within the tongue environment is thus performed by different taxa outside of the *Neisseriaceae* family. Furthermore, contrary to previous findings (11, 48, 49), our study revealed that *N. mucosa* is not the major nitrate reducer in dental plaque; instead, this function is likely carried out by *N. oralis* and *N. elongata*. While many *N. mucosa* genomes possess a complete nitrate reductase operon (NarGHIJ), we found that the most common sub-group of *N. mucosa* lacks these genes. In contrast, all genomes of *N. oralis* and *N. elongata*, both species prevalent in dental plaque, possess the complete nitrate reductase operon. These findings refine our understanding of nitrate reduction in the oral cavity, highlighting the key roles of *N. oralis* and *N. elongata* in this process.

The ability to metabolize diverse nutrient sources may be a key adaptation for plaque specialists, enabling them to thrive in the nutrient-limited and highly competitive dental plaque environment. Our findings highlight several metabolic pathways that enhance nutrient versatility in plaque specialists, including lysine degradation, aromatic compound utilization, and sulfur metabolism. Notably, plaque specialists are enriched for the lysine degradation pathway, which allows bacteria to produce succinate from D-lysine. This pathway is particularly advantageous, as plaque specialists possess the amino acid racemase RacX, enabling the conversion of D-lysine to L-lysine and thereby utilizing both stereoisomers of lysine as carbon and energy sources, or potentially as a component of peptidoglycan (50). Such metabolic flexibility may confer an edge in environments where nutrient availability fluctuates.

Adhesion is crucial for the colonization and persistence of *Neisseriaceae* species in the oral environment. Our study indicates that plaque specialists are enriched in adhesion mechanisms compared to tongue and keratinized gingiva specialists, including proteins involved in fimbriae and pilus assembly. Studies show that the adhesion capabilities of *Neisseria*, *Kingella*, *Eikenella*, and *Simonsiella* spp. are critical for their colonization and pathogenicity. For instance, *Neisseria meningitidis* uses type 4 pili to adhere to and penetrate the human nasopharyngeal epithelium, a process essential for colonization and potential invasion (12, 51, 52). *Kingella kingae* utilizes type 4 pili and other surface proteins to adhere to and invade epithelial cells in the respiratory tract, contributing to its pathogenicity in pediatric septic arthritis and osteomyelitis (53). *Eikenella corrodens*, a member of the HACEK group, adheres to epithelial cells and the extracellular matrix through pili and outer membrane proteins. This adhesion capability plays a critical role in its involvement in infections such as periodontitis and infective endocarditis, especially in immunocompromised individuals (54). The adhesion mechanisms of *Simonsiella* spp. are less well characterized. However, they are known to contribute to the microbial diversity of the oral ecosystem by adhering to various surfaces within the oral cavity, potentially utilizing similar adhesin strategies as other *Neisseriaceae* members (11). These diverse and specialized adhesion mechanisms underscore the ability of *Neisseriaceae* to colonize specific niches within the human body and their role in health and disease. Insights into these adhesion mechanisms could pave the way for targeted therapeutic interventions to prevent infections associated with pathogenic bacteria.

Our findings indicate that a limited number of antimicrobial resistance genes, such as beta-lactamase PenP, are associated with specific habitat preferences among oral *Neisseriaceae*. However, the distribution of these genes does not strictly follow phylogenetic lines or habitat types. This irregular distribution suggests that factors beyond vertical inheritance, such as horizontal gene transfer (HGT), may contribute to the presence of PenP in distantly related taxa. HGT has played a key role in the spread of β-lactam resistance from commensal to pathogenic *Neisseria* (55–59), raising the possibility that similar mechanisms facilitate resistance gene exchange among oral commensals. Future research should examine the interplay between HGT, selective pressures, and species-specific constraints to better understand how resistance genes are maintained and disseminated in oral microbial communities.

A striking observation in our mapping results was the frequent dominance of a single species in individual oral samples. Here, we propose several hypotheses to explain how this pattern could arise. One hypothesis is that species such as *N. mucosa*, *N. elongata*, and *K. oralis* compete for the same niche, and their interactions are driven by antagonistic mechanisms. An example of such a mechanism is the production of toxins to inhibit related species, such as the inhibition of *N. meningitidis* by MafB toxins produced by *N. lactamica* (60). MafB-associated genes are present in the oral cavity in *N. mucosa* but not in the other species, hinting at MafB-mediated competition as a potential driver of niche dominance of *N. mucosa* in plaque samples. Alternatively, *N. mucosa*, *N. elongata*, and *K. oralis* could each be specialized for a distinctive microhabitat within plaque, and the abundance of each taxon in a given plaque sample could be a consequence of patchy distribution of these microhabitats. Other oral bacteria are known to specialize for microhabitats within plaque (61), and oral biofilms are inhomogeneous, with patches dominated by one or a few taxa (62, 63). Further research is needed to disentangle these factors and better understand the drivers of microbial dominance in this habitat.

In conclusion, this study provides a comprehensive analysis of the ecological niches and adaptive strategies of *Neisseriaceae* species within the human oral microbiome. Metagenomic mapping revealed the distribution of these bacteria, and pangenomic analysis identified key genes and pathways that may drive their habitat preferences. These findings contribute to a broader understanding of the impacts of *Neisseriaceae* on oral health and disease and pave the way for future research and therapeutic interventions.

## Data Availability

The raw data used in this study are publicly available at National Institutes of Health GenBank and RefSeq (https://www.ncbi.nlm.nih.gov/genome/) for genomes and Human Microbiome Project metagenomes from https://portal.hmpdacc.org/. The code used for analyses is available on GitHub (https://github.com/FatherofEverest/Spatial-ecology-of-the-Neisseriaceae-family-in-the-human-oral-cavity).

## SUPPLEMENTAL MATERIAL

Fig. S1 spectrum.03275-24-s0001.pdf Identification of sub-species level subgroups within *Neisseria subflava major clades.*

Fig. S2 spectrum.03275-24-s0002.pdf Identification of sub-species level subgroups within *Neisseria mucosa* major clades.

Fig. S3 spectrum.03275-24-s0003.pdf Occurrence of KEGG metabolic pathway modules across *Neisseriaceae* genomes and by habitat preference.

Fig. S4 spectrum.03275-24-s0004.pdf Occurrence of COG20 functions across *Neisseriaceae* genomes and by habitat preference.

Supplemental material spectrum.03275-24-s0005.docx Supplemental text and supplemental figure legends, providing further details of results for Pangenome, Phylogeny, Average Nucleotide Identity, and comparisons with GTDB.

Tables S1 to S4 spectrum.03275-24-s0006.xlsx Supplemental tables.

Tables S5 to S8 spectrum.03275-24-s0007.xlsx Supplemental tables.

Tables S9 to S16 spectrum.03275-24-s0008.xlsx Supplemental tables.

ASM does not own the copyrights to Supplemental Material that may be linked to, or accessed through, an article. The authors have granted ASM a non-exclusive, world-wide license to publish the Supplemental Material files. Please contact the corresponding author directly for reuse.
