# Supplementary figures and images for: Spatial ecology of the Neisseriaceae family in the human oral cavity
Source: Microbiol Spectr. 2025 Apr 8;13(5):e03275-24. doi: 10.1128/spectrum.03275-24 (PMC12054151; doi:10.1128/spectrum.03275-24)

A

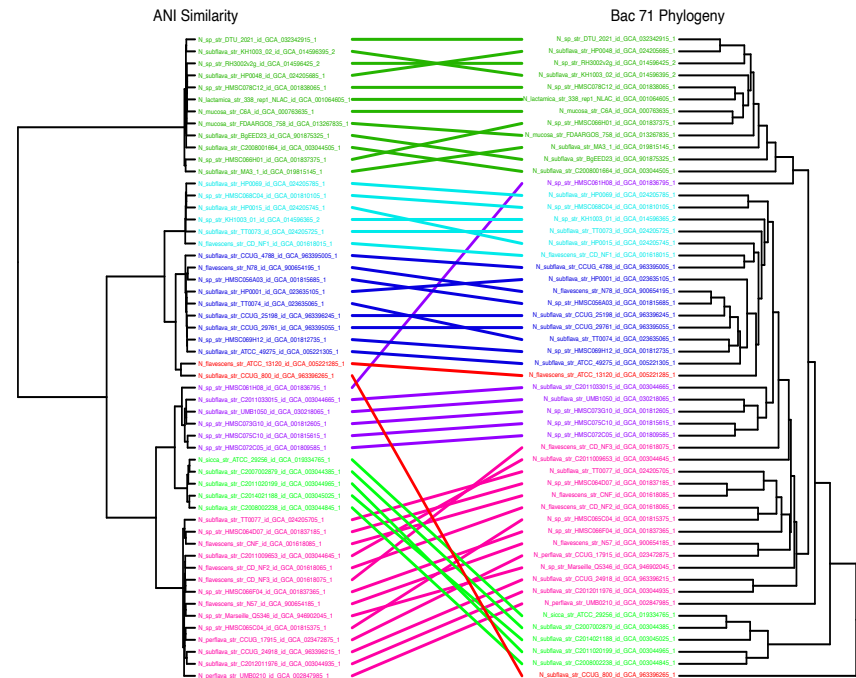

B

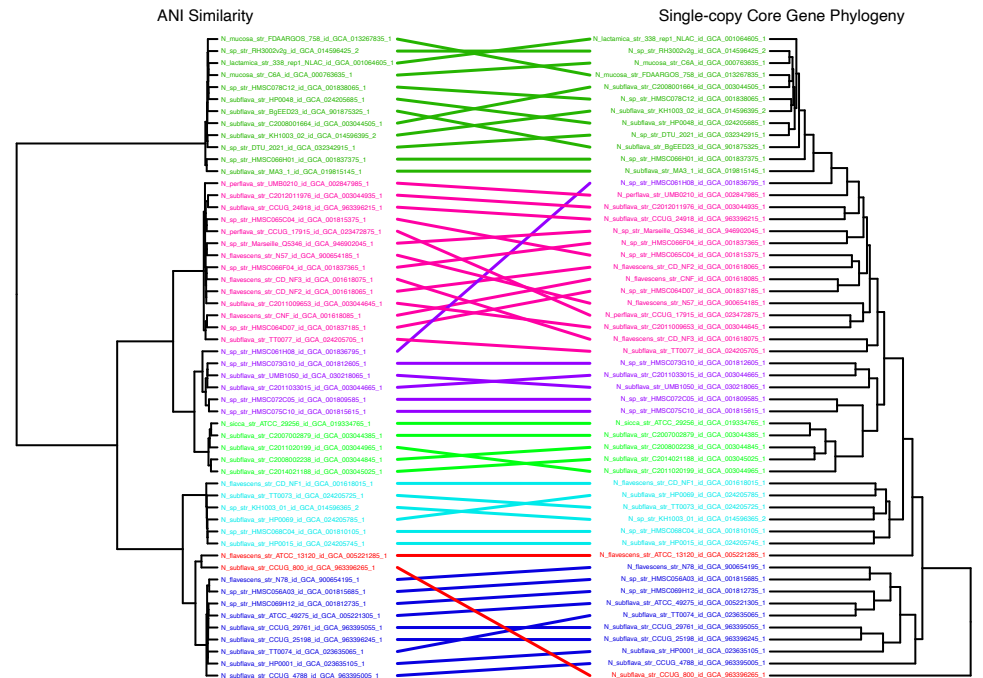

C

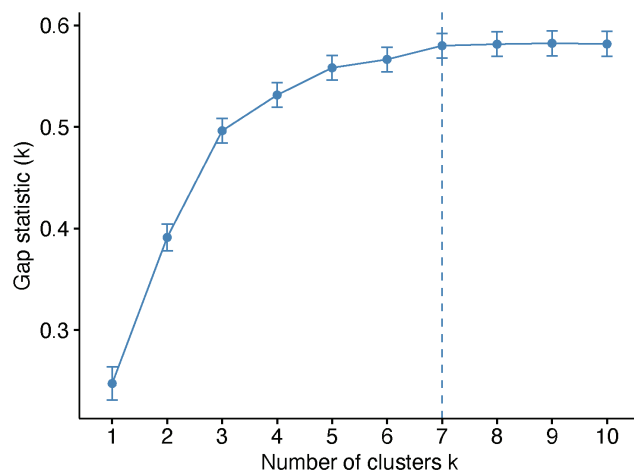

D

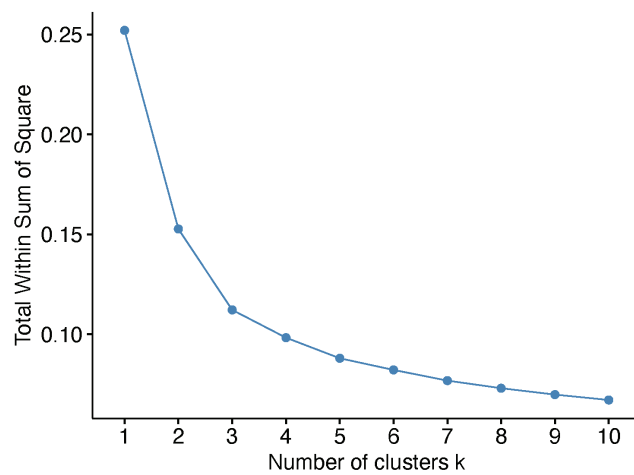

E

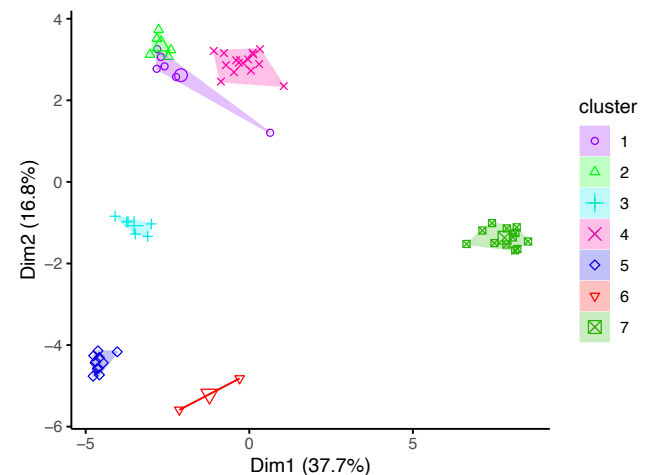

Supplement: Fig. S1 — Identification of sub-species level subgroups within Neisseria subflava major clades. [file spectrum.03275-24-s0001.pdf]

A

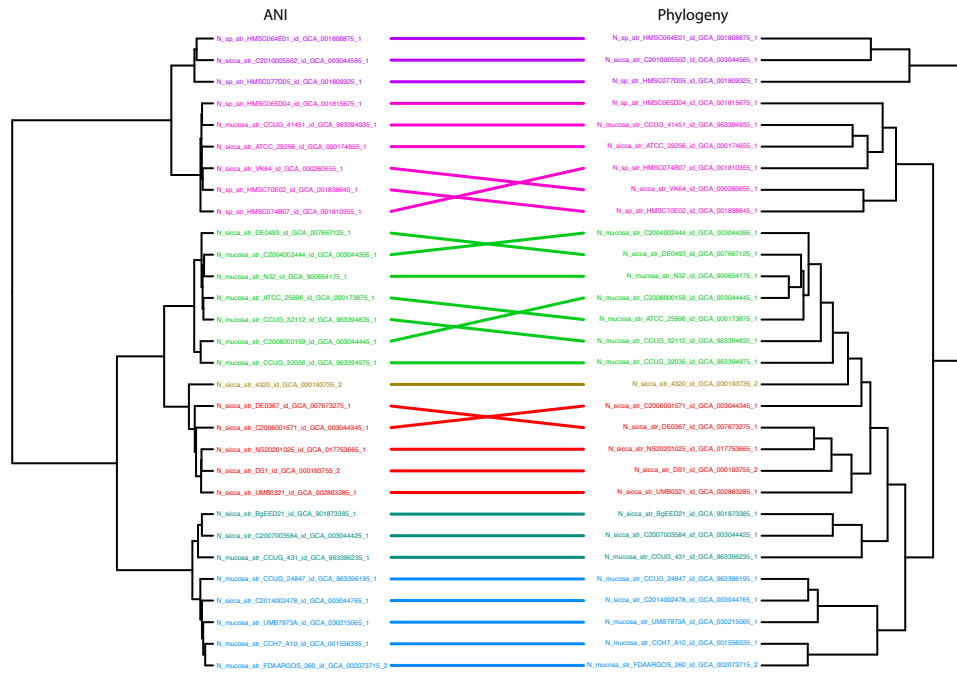

B

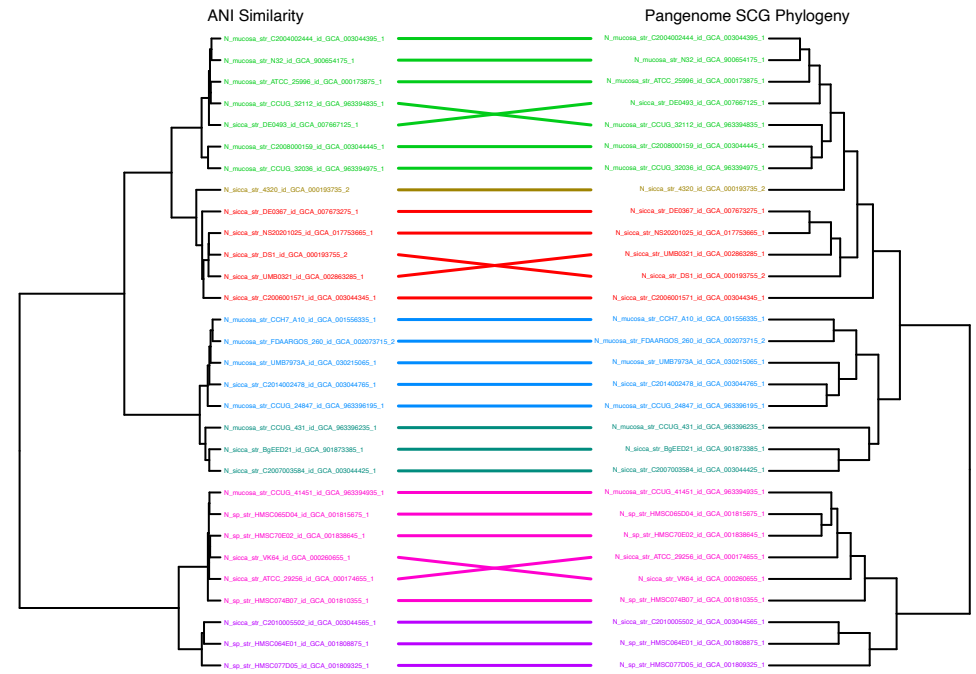

C

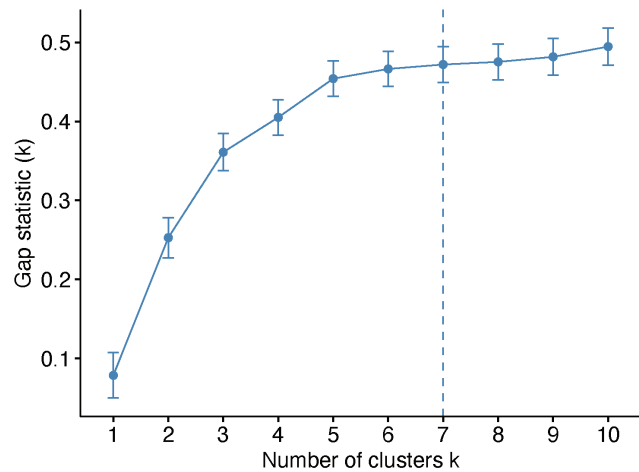

D

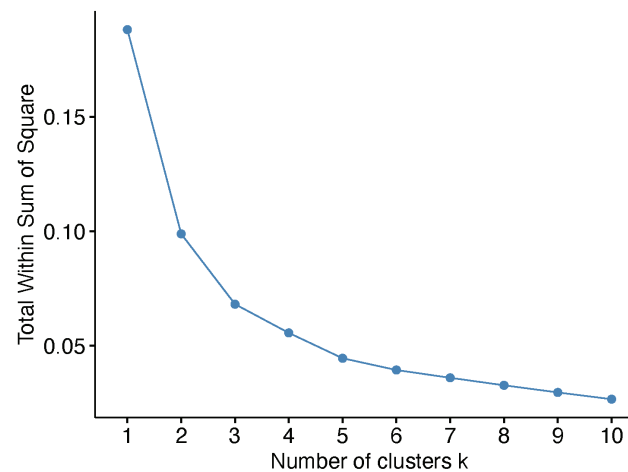

E

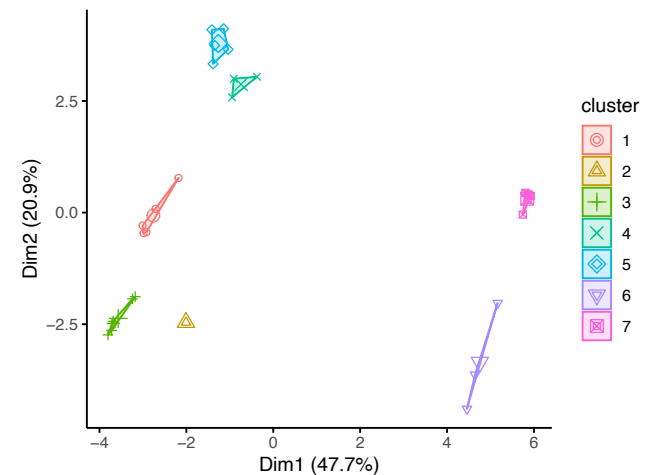

Supplement: Fig. S2 — Identification of sub-species level subgroups within Neisseria mucosa major clades. [file spectrum.03275-24-s0002.pdf]

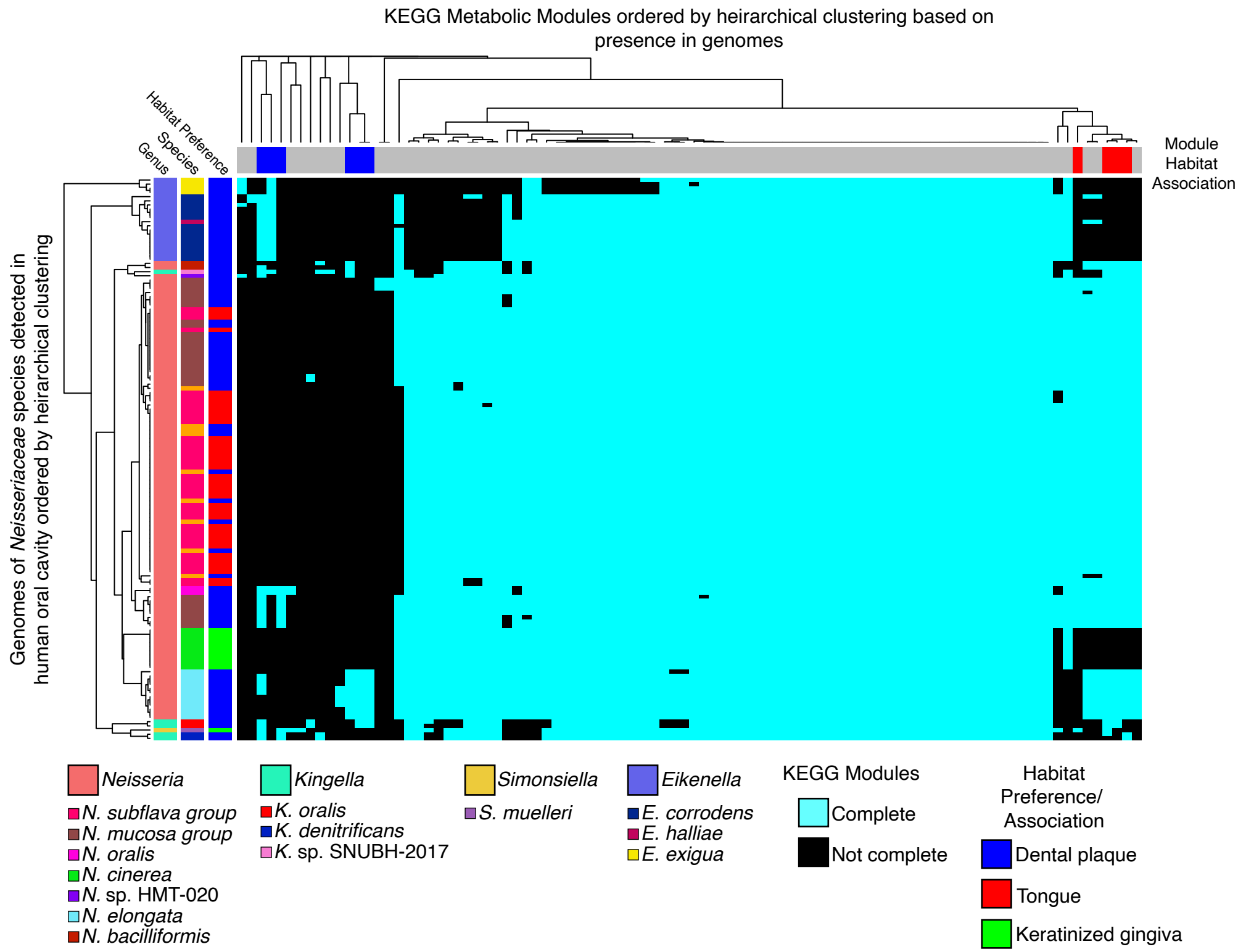

Supplement: Fig. S3 — Occurrence of KEGG metabolic pathway modules across Neisseriaceae genomes and by habitat preference. [file spectrum.03275-24-s0003.pdf]

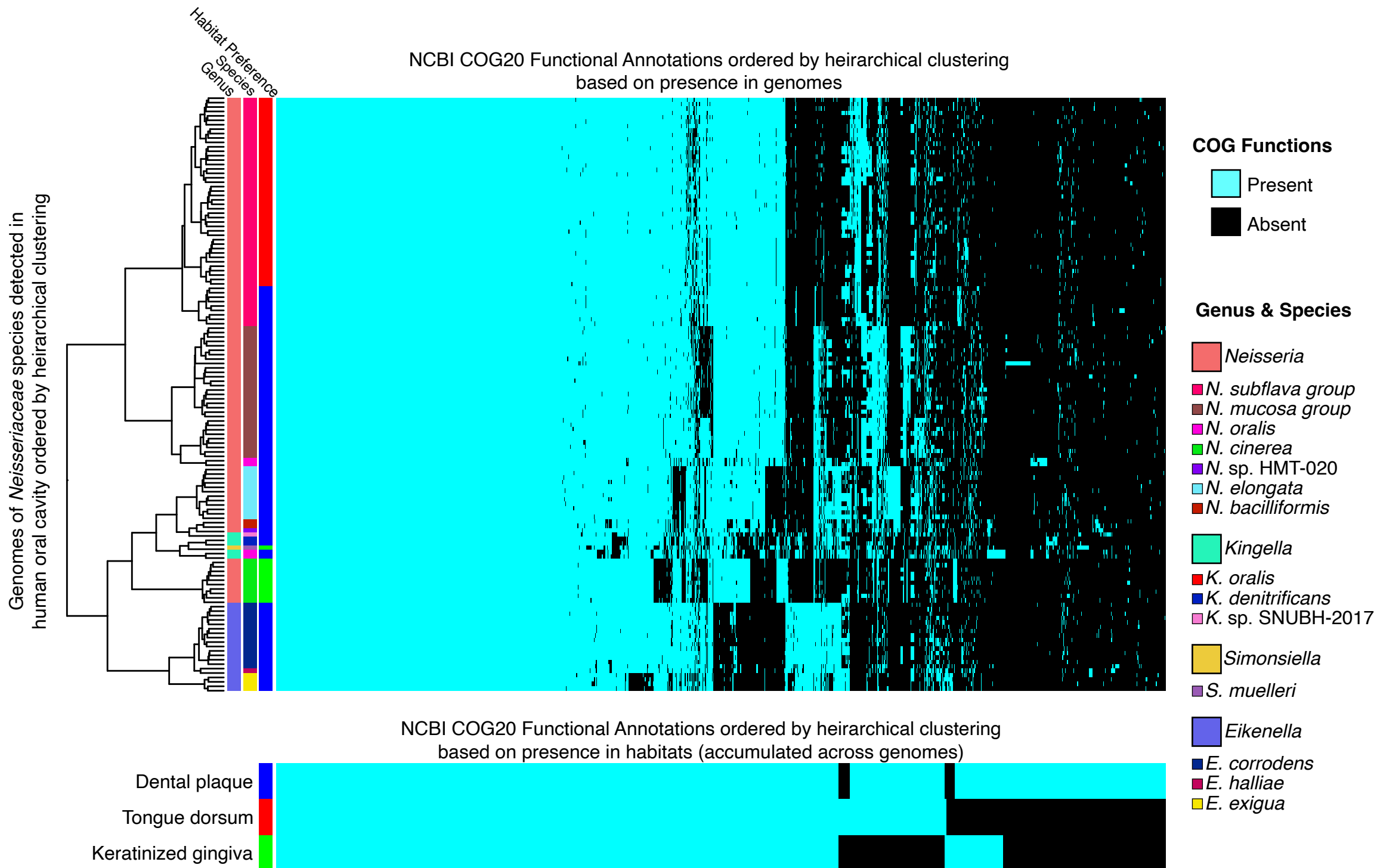

Supplement: Fig. S4 — Occurrence of COG20 functions across Neisseriaceae genomes and by habitat preference. [file spectrum.03275-24-s0004.pdf]
