# Supplementary material for: Spatial ecology of the Neisseriaceae family in the human oral cavity
Source: Microbiol Spectr. 2025 Apr 8;13(5):e03275-24. doi: 10.1128/spectrum.03275-24 (PMC12054151; doi:10.1128/spectrum.03275-24)
Supplement: Supplemental material — Supplemental text and supplemental figure legends, providing further details of results for Pangenome, Phylogeny, Average Nucleotide Identity, and comparisons with GTDB. [file spectrum.03275-24-s0005.docx]

SUPPLEMENTAL MATERIALS

Title: Spatial ecology of the *Neisseriaceae* family in the human oral cavity

Jonathan J. Giacomini^1,4^, Julian Torres-Morales^1^, Floyd E*.* Dewhirst^1,2^, Gary G. Borisy^1^, Jessica L. Mark Welch^1,3^

^1^ADA Forsyth Institute, Cambridge, MA 02142

^2^Harvard School of Dental Medicine, Boston, MA 02115, USA.

^3^Marine Biological Laboratory, Woods Hole, MA 02543, USA

**Supplemental Text:**

***Pangenome, Phylogeny, Average Nucleotide Identity and comparison with GTDB:***

The *Neisseria* group showed a complex set of relationships. One observation was the polyphyletic nature of both the *N. subflava* and *N. mucosa* major clades, each including genomes from multiple named species. The *N. subflava* major clade included genomes from species recognized by NCBI as *N. subflava*, *N. flavescens*, *N. perflava*, 16 *N.* sp. genomes, and potentially misclassified genomes from *N. sicca*, *N. mucosa*, and *N. lactamica*. The *N. mucosa* major clade included *N. sicca*, *N. mucosa*, and 5 *N.* sp. genomes. Both the *N. subflava* and *N. mucosa* major clades contained intraspecies ANI values below the 95% threshold, indicating the presence of distinct subgroups. Tanglegrams and results from hierarchical clustering analyses revealed distinct subgroups for both the *N. subflava* and *N. mucosa* major clades (Figures S1 and S2, Supplemental Data: Table S6). The *Neisseria* sp. genome (Strain F0370), designated HMT 020 in the Human Oral Microbiome Database (HOMD) and isolated from the human oral cavity shares less than 80% ANI with any other genome in the reference set and therefore appears more distantly related in phylogeny. Several apparent misclassifications were notable, likely representing misnamed genomes in NCBI. These include a single *N. cinerea* genome (Strain CCUG 5746) clustered within the *N. polysaccharea* clade and a single *N. lactamica* genome (Strain NS19) clustered within the *N. polysaccharea* clade. The analysis also revealed distinct clustering patterns for several non-human, animal-associated *Neisseria* species. Interestingly, the pangenome grouped non-human-associated *Neisseria* genomes into one distinct clade that included *Neisseria* *oralis.*

The *Eikenella* genus showed distinct clustering patterns across different measures of genetic relatedness. The *E. corrodens* genomes, including strains EI_09, NML130388, and CC92I, consistently grouped together, supporting their close evolutionary relationship. The high ANI values among *E. corrodens* strains reinforced their classification as a single species. However, a single *E. corrodens* genome (Strain EI_05) clustered with the *E. halliae* genome (Strain NML130454) in both the pangenome and phylogeny, sharing 94.2% ANI. *Eikenella* *exigua* (strains PXX and EI_02) displayed genetic distinctness from *E. corrodens*, indicating species-level differentiation. Additionally, *E.* *halliae* (strain NML130454) and *Eikenella* *glucosivorans* (strain S3360) each formed separate clusters, highlighting their unique genetic identities within the genus. The overall phylogenetic and pangenomic analyses of the *Eikenella* genus reveal a well-defined genomic structure, with clear distinctions between genomes of *E. corrodens*, *E.* exigua, *E. halliae*, and *E. glucosivorans*, providing validation for their classification as distinct species.

Our analyses revealed that the *Kingella* genus is both paraphyletic and polyphyletic, indicating that its current taxonomic classification does not fully reflect the evolutionary relationships within the genus. Specifically, *K. potus* appears to be distinct from the other species in the *Kingella* genus and is more closely related to *N. bacilliformis* than to other *Kingella* species. (Figure 2B, Supplemental Data: Table S7). The *K. oralis* genome (Strain ATCC 51147) clustered with the newly identified *K. bonacorsii* genome (Strain Marseille Q4569) in all three measures of genetic relatedness, sharing 95.7% ANI and, therefore, likely represents the same species. Our phylogenetic analyses and pangenome clustering underscore the complexity of the *Kingella* genus, highlighting the need for a re-evaluation of its taxonomic classification to better reflect the evolutionary relationships among these species.

**Supplemental Figure Legends:**

**Figure S1**: Identification of sub-species level subgroups within *Neisseria subflava* major clades. Panels (A-E) represent various aspects of the sub-species level analysis of *Neisseria subflava*. Panel A shows a tanglegram comparing Average Nucleotide Identity (ANI)-based hierarchical clustering with the phylogeny derived from the universal set of 71 bacterial single-copy core genes (SCCGs), illustrating the congruence between ANI-based and phylogenomic subgroup classifications. Panel B presents a tanglegram comparing ANI-based hierarchical clustering with the phylogeny based on SCCGs extracted from the *N. subflava* pangenome, highlighting similarities and differences in subgroup delineations between ANI and pangenome SCCGs. Panel C shows the results of the Gap statistic used to determine the optimal number of clusters (k) based on ANI similarity. Panel D depicts the total within-cluster sum of squares as a function of the number of clusters (k), which aids in selecting the appropriate number of clusters for ANI-based grouping. Panel E provides a Principal Component Analysis (PCA) plot showing the distribution of *N. subflava* genomes based on ANI, highlighting potential clustering patterns and genetic diversity within the major clades.

**Figure S2**: Identification of sub-species level subgroups within *Neisseria mucosa* major clades. Panels (A-E) represent various aspects of the sub-species level analysis of *Neisseria mucosa*. Panel A shows a tanglegram comparing ANI-based hierarchical clustering with the phylogeny derived from the 71 universal bacterial SCCGs, depicting the relationships between subgroups inferred by each method. Panel B presents a tanglegram comparing ANI-based hierarchical clustering with the SCCG phylogeny extracted from the *N. mucosa* pangenome, demonstrating how ANI and pangenomic approaches align or diverge in defining sub-species groups. Panel C shows the results of the Gap statistic used to determine the optimal number of clusters (k) for ANI similarity. Panel D depicts the total within-cluster sum of squares for each possible number of clusters, used to evaluate the optimal number of clusters for *N. mucosa* based on compactness. Panel E provides a Principal Component Analysis (PCA) plot showing the distribution of *N. mucosa* genomes based on ANI, highlighting potential clustering patterns and genetic diversity within the major clades.

**Figure S3**: Occurrence of KEGG metabolic pathway modules across Neisseriaceae genomes and by habitat preference. This figure presents a comparative binary map visualization of KEGG modules detected in 136 *Neisseriaceae* genomes isolated from the human oral cavity. The binary map displays the presence/absence of 92 KEGG modules detected at a 75% completeness threshold across individual genomes of detected *Neisseriaceae* species. The columns represent different KEGG modules, while the rows correspond to the genomes of the detected species. Hierarchical clustering was applied to both rows and columns using the complete method with Jaccard distance to reveal patterns of shared modules between genomes. Genera and species are distinguishable based on the presence or absence of their KEGG modules, reflecting distinct genetic adaptations. Along the top of the binary map, a color bar indicates a habitat association of each module determined from a statistical enrichment test. Gray modules were not significantly (q value > 0.01) associated with a habitat. This clustering allows for a habitat-based comparison of functional capabilities across different ecological niches within the oral cavity.

**Figure S4**: Occurrence of COG20 functions across Neisseriaceae genomes and by habitat preference. This figure presents a comparative binary map visualization of COG20 functional annotations detected in 136 *Neisseriaceae* genomes isolated from the human oral cavity. The figure is organized as two panels: (Top) Displays the presence/absence of 2,294 detected COG20 functions across individual genomes of detected *Neisseriaceae* species. The columns represent different COG functions, while the rows correspond to the genomes of the detected species, ordered based on hierarchical clustering. Clustering was performed using Ward's method with Euclidean distance. Genera and species are distinguishable based on the presence or absence of their COG20 functional annotations, reflecting distinct genetic adaptations. (Bottom) Shows the presence of COG20 functions aggregated by habitat preference (plaque, tongue dorsum, and keratinized gingiva). A function is marked as present in a habitat if at least one genome from that habitat possesses the function. Hierarchical clustering was applied to both rows and columns using Ward's method with Euclidean distance to reveal patterns of shared gene functions between habitats. This clustering allows for a habitat-based comparison of functional capabilities across different ecological niches within the oral cavity.
